# Dancing with the Devil: A Review of the Importance of Host RNA-Binding Proteins to Alphaviral RNAs during Infection

**DOI:** 10.3390/v15010164

**Published:** 2023-01-05

**Authors:** Claire E. Westcott, Cierra M. Isom, Deepa Karki, Kevin J. Sokoloski

**Affiliations:** 1 Department of Microbiology and Immunology, School of Medicine, University of Louisville, Louisville, KY 40202, USA; 2 Center for Predictive Medicine for Biodefense and Emerging Infectious Disease (CPM), University of Louisville, Louisville, KY 40202, USA

**Keywords:** alphavirus, RNA-binding protein, host/pathogen interactions

## Abstract

Alphaviruses are arthropod-borne, single-stranded positive sense RNA viruses that rely on the engagement of host RNA-binding proteins to efficiently complete the viral lifecycle. Because of this reliance on host proteins, the identification of host/pathogen interactions and the subsequent characterization of their importance to viral infection has been an intensive area of study for several decades. Many of these host protein interaction studies have evaluated the Protein:Protein interactions of viral proteins during infection and a significant number of host proteins identified by these discovery efforts have been RNA Binding Proteins (RBPs). Considering this recognition, the field has shifted towards discovery efforts involving the direct identification of host factors that engage viral RNAs during infection using innovative discovery approaches. Collectively, these efforts have led to significant advancements in the understanding of alphaviral molecular biology; however, the precise extent and means by which many RBPs influence viral infection is unclear as their specific contributions to infection, as per any RNA:Protein interaction, have often been overlooked. The purpose of this review is to summarize the discovery of host/pathogen interactions during alphaviral infection with a specific emphasis on RBPs, to use new ontological analyses to reveal potential functional commonalities across alphaviral RBP interactants, and to identify host RBPs that have, and have yet to be, evaluated in their native context as RNA:Protein interactors.

## 1. Introduction

Alphaviruses are single-stranded positive sense RNA viruses that are transmitted to vertebrate hosts primarily by mosquitoes, and thus are referred to as arthropod-borne viruses or arboviruses [1,2]. Large scale outbreaks of alphaviruses were first reported in the 1920′s and have happened every decade since, becoming increasingly larger and more frequent [3,4,5]. This is, at least in part, due to the expansion of mosquito populations to new areas because of climate change and human interventions [6]. Belonging to the family Togaviridae, the alphaviruses are classified into two groups, specifically as either the Old World (OW) and the New World (NW) alphaviruses, based on their geographic location and relative distributions. In addition, the Old World and New World viruses differ regarding the disease manifestations they cause. Along with the initial febrile illness, the OW alphaviruses, such as Chikungunya virus (CHIKV), Semliki Forest Virus (SFV), and Ross River Virus (RRV), can cause debilitating arthritis in multiple joints, which persists months to years after the resolution of acute infection [7,8,9]. Sindbis Virus (SINV) is the infectious agent for diseases like Pogosta, Ocklebo, and Karelian fever, all of which are hallmarked by severe arthralgia-like disease; however, phylogenetic analyses indicate that it is genetically similar to the NW alphaviruses [10,11,12]. Infections of the encephalitic NW viruses Venezuelan Equine Encephalitis Virus (VEEV), Eastern Equine Encephalitis Virus (EEEV), and Western Equine Encephalitis Virus (WEEV) exhibit higher mortality despite being comparatively rarer clinically and can cause severe neurological sequelae in those that survive encephalitic infection [13].

Like most RNA viruses, alphaviruses rely on host proteins and machinery to complete their viral lifecycles. This includes using the host proteins and machinery to functionalize the RNAs to enable the translation of the viral RNAs (vRNAs) and the final assembly of mature viral particles at the conclusion of the life cycle [14,15,16]. For over 30 years, there have been many studies conducted with the goal of determining what host proteins interact with alphaviruses during infection. Though these studies all look at host protein interactions by way of different methods, host RNA binding proteins (RBPs) have consistently emerged as viral interactants. As such, it is important to comprehensively examine the discovery efforts of host RNA binding proteins interacting with alphaviruses, analyze the RBP interactants via ontological analyses to identify areas of enrichment, and discuss specific roles of RBPs during alphaviral infection to illuminate the importance of RBPs to alphaviral infections and identify where future research is warranted.

## 2. Alphavirus Background

### 2.1. An Overview of the Molecular Lifecycle

**Figure 1:**
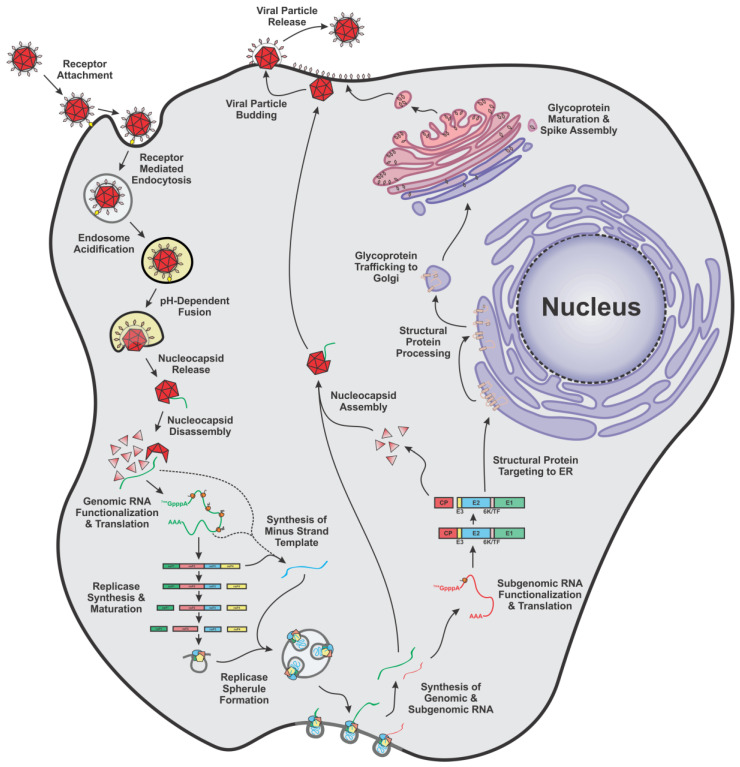
A Schematic Diagram of the Alphavirus Lifecycle. A simplified schematic diagram of the major lifecycle events as noted in the text.

As shown in Figure 1, alphavirus infection begins as the viral E2 glycoproteins, which are prominently displayed on the outside of the virion as a trimeric spike of heterodimers of E1 and E2, bind to the receptor that is on the host cell membrane. The specific cell host receptor(s) depends on the alphaviral species and host cell, and there can be multiple receptors for one alphavirus species [2,17]. Interaction with the receptor results in viral uptake into the cell by receptor-mediated endocytosis [18,19]. As the virus-containing endosome matures, it acidifies, which is critical for a major conformational change of the glycoproteins, and the viral particle undergoes fusion with the endosomal membrane via the fusion peptide of the E1 glycoprotein [20,21,22]. The result is the release of the nucleocapsid core into the host cytoplasm, after which the newly delivered nucleocapsid core rapidly disassembles and releases the vRNA for translation [23]. The precise mechanism with which disassembly of the core and the subsequent functionalization of the vRNA for protein translation is not well understood; however, host protein engagement and relocalization of the vRNA to the ribosomes are known to be critical for a successful lifecycle [24,25]. First, after the release and functionalization of the viral RNA, the alphaviral nonstructural polyprotein, P123, is translated from the newly bare vRNA as well as a P1234 polyprotein due to slippage at the Opal stop codon (however, not all alphaviruses possess an Opal stop codon, and rather produce P1234 as the native polyprotein) [26,27]. After synthesis, the nsP2 component of the polyprotein (and individually in isolation as a monomer) proteolytically cleaves off the nsP4 protein [28]. The resulting polyprotein P123 and the RNA-dependent RNA Polymerase (RdRp) nsP4 form to make the initial replicase complex that synthesizes the minus strand RNA, which serves as the template for replication and transcription of the positive-sense vRNAs [29]. After the synthesis of as little as one minus strand RNA, the P123 component of the replicase complex is further processed as nsP1 is cleaved off to generate the short-lived nsP1-P23-nsP4 complex [28,30]. After P23 is processed in *trans* by another nsP2 protein or nsP2-containing polyprotein, the four nsP proteins together form the fully mature replicase complex that synthesizes both the genomic and subgenomic strand, but not the minus strand [28,30,31,32,33,34]. This viral RNA synthesis occurs in invaginated spherules derived from host membranes; however, the specific site of the spherules is dependent on the particular alphaviral species [35,36]. The structural polyprotein is translated from the subgenomic RNA strand in the order of Capsid-pE2-6K/TransFrame-E1. Like the nonstructural polyprotein, the structural polyprotein is proteolytically processed into monomeric proteins during infection; however, unlike the nonstructural polyprotein, the structural proteins are processed by viral and host proteases to complete their maturity [16,37]. After the capsid (CP) protein is translated, it autoproteolytically cleaves itself off from the actively translating structural polypeptide, inactivating the protease activity of the CP protein in the process [32]. The monomeric CP protein is then free to interact with the gRNA, and other CP proteins, to form the nascent nucleocapsid core [15]. Consequentially, the leftover structural polyprotein contains a signal sequence that targets the structural polypeptide to the ER for further translation and processing where the structural polyprotein is then cleaved by host proteases like signalase and furin to create the monomeric structural protein precursors [38,39,40,41]. Several of the structural components are then post-translationally modified, including the post-translational modifications of the glycoproteins and TransFrame (TF) protein [42,43,44,45,46,47]. After being glycosylated, palmitoylated, and rearrangement of the disulfide bonds, the glycoproteins are then trafficked and displayed on the host cell surface. The CP protein of the nucleocapsid core and the cytoplasmic endodomain of the E2 protein then interact, and this is thought to be a major driver of the budding of the newly formed virions from the infected host cell [48,49,50].

### 2.2. Genetic Organization of Alphaviruses

**Figure 2:**
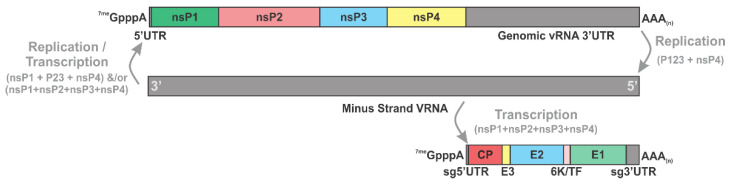
Organization of the Alphavirus Viral RNAs. Schematic diagrams of the coding organization of the SINV Genomic (top), Minus (middle), and Subgenomic (bottom) RNAs. The individual components of the nonstructural and structural polyproteins are indicated as are the untranslated regions (UTRs) and major features of the RNAs. Individual components are drawn to scale, relative to one another. Viral RNA synthetic processes responsible for the synthesis of each vRNA species, and the replicase complexes primarily responsible for their synthesis are shown in grey.

Alphaviruses produce three vRNA species during infection; the minus strand, the genomic strand, and the subgenomic strand, as demonstrated in Figure 2. Of these, the genomic and negative strands are replicated, and the genomic and subgenomic strands are translated. In total, the viral genome of a typical alphavirus is about 11.5 kb long, which often, but not always, begins with a type-0 ^7-me^GpppA cap and ends with a 3′ poly(A) tail [51,52,53]. The alphavirus genomic RNA (gRNA) has two coding regions, of which only the first acts as an open-reading frame (ORF) in the context of the gRNA, which encodes the nonstructural proteins that create the viral replicase complex. The second ORF translates from a separate subgenomic RNA (sgRNA) and contains the coding information for the structural proteins that will make up the virion. The minus strand RNA is made from the full-length gRNA by the P123-nsP4 replicase complex, and as this RNA is a negative-sense copy of the gRNA it acts as the replication template for the synthesis of the other viral RNA species.

Alphaviruses have several sequences and RNA structures in coding and non-coding regions that are essential for a productive lifecycle. Both 5′ untranslated regions (UTRs) of the gRNA and sgRNA are highly structured, which is believed to allow for the recruitment of host and viral proteins required for efficient replication and translation [54,55,56]. The genomic 5′ UTR and the complementing negative strand 3′ UTR contain core promoter elements that are critical for plus and minus-strand RNA synthesis [57,58,59]. Near the beginning of the coding region of nsP1, the 5′ end also contains a conserved sequence element (CSE) that is important for replication in mosquitoes but not the mammalian host [60]. The 3′ UTR of the alphaviruses is generally much longer than the 5′ UTR proper, and it has several key components that are important to lifecycle and infection [61]. It is important to note that the functional 3′UTR of the gRNA is different from that of the sgRNA, as the entire structural coding region is translationally silent in the context of the genome. Regarding the common gRNA and sgRNA 3′UTR sequences, most alphaviruses have, at minimum, a series of repeated sequence elements (RSE) and a CSE, with the amount of RSEs varying from 2–5, depending on the specific viral species [61]. The 3′ tail end is also important in host protein interactions, which is discussed in detail later in the third section of this review.

## 3. Discovery Efforts

As with many viruses, the identification of host/pathogen interactions has been a topic of significant interest. The earliest published reports describing specific alphaviral host/pathogen interactions were those of the Strauss lab, where discrete host proteins (later determined to include the host La protein) were observed binding to the alphaviral RNAs during infection [62,63]. Since these foundational studies, significant efforts to understand and appreciate the full scope the alphaviral host/pathogen interface have led to the identification of many host factors involved in alphaviral biology. In this section, we aim to provide a concise summary of studies that have contributed to the understanding of alphaviral host/pathogen Protein:Protein (P:P) and RNA:Protein (R:P) interactions.

### 3.1. Defining the Protein:Protein Interaction Interface of the Alphaviruses with their Host

Broadly speaking, there have been three primary methodologies employed to define the P:P interactions of the alphaviral nonstructural proteins (nsPs) individually or in the context of the replication complex. The earliest and most common method relies on the incorporation of reporter proteins or epitope tags into the target nsPs to enable the capture of interaction partners for subsequent identification via mass spectrometry [64,65,66,67]. Alongside the immunoaffinity purification approaches, functional replication complexes have been purified from cell lysates using magnetic fractionation to enable the identification of host factors engaging with or associated alongside the replication complex [68]. In addition to these two purification-based approaches, a yeast two-hybrid based approach has been used to examine the P:P interactions of the alphaviral nsPs [69]. Altogether, these efforts have established the knowledgebase regarding the host/pathogen P:P interaction interface.

As introduced above, an early primary strategy by which the alphaviral nsP P:P interactions were identified was using immunoaffinity purification. This specific approach was used by the Frolova/Frolov and MacDonald groups to probe the P:P interactions of the nsP3 protein via a series of contemporary efforts [64,65]. That efforts to identify the interactants of the nsP3 protein were at the forefront of identifying alphaviral nsP P:P interactions, which is unsurprising; the prioritization of nsP3 was likely due to a combination of convenience and mystique, as the nsP3 protein is more forgiving to the inclusion of exogenous sequences and the functional importance of the nsP3 protein during infection has remained enigmatic for many years. Both groups employed the use of SINV strains containing a Green Fluorescent Protein (GFP) reporter in the nsP3 ORF to enable the purification of nsP3 complexes via anti-GFP antibody immunoprecipitation. Nonetheless, despite using similar approaches, the control conditions differed between these two studies to a significant extent. Frolova et al. 2006 used comparative purifications of infected and uninfected lysates to select and identify putative interactants [64], whereas Cristea et al. (2006) utilized a “free” GFP control, where the GFP reporter was expressed independently of nsP3, to allow for the selection and discriminative assignment of host factor interactions following immunoaffinity purification [65]. Regardless, these efforts led to the identification of approximately 30 and 35 host factors, respectively, 10 of which were common between the two nsP3 studies including, most notably, the host G3BP1/2 proteins. In addition to examining the P:P interactions of the nsP3 protein, this immunoaffinity purification approach was also used by the Frolova group to examine the P:P interactions of the nsP2 protein [66]. These efforts resulted in the detection of host factors unique to nsP2 and many factors that were previously observed during the studies involving nsP3. These observations led them to postulate that these host factors may be interacting with multiple nonstructural proteins or be integral parts of the replicase complex. This hypothesis was largely confirmed by later work from Varjak et al. (2013) (as described below) [68]. Finally, the P:P interactions of the nsP4 protein were assessed by the MacDonald group, as described by Cristea et al. (2010); however, this method relied on the incorporation of the smaller FLAG-tag epitope as the inclusion of GFP in the nsP4 protein was incompatible with nsP4 function [70]. From these efforts, nsP4 was similarly found to interact with unique and common host proteins, as shown above.

Together with the immunoaffinity purification-based approach, a magnetic fractionation approach has been used to isolate and study the replication compartments of SFV for the purpose of identifying host/pathogen P:P interactions [68]. As reported in Varjak et al. 2013, gel-free mass spectrometric analysis of the replicase complexes resulted in the identification of 78 distinct host factors. While these efforts revealed many novel host interactants, many factors observed as co-associating with the SFV replicase complexes had been previously identified through the immunoaffinity purification approaches described above. Regardless, to date, these efforts represent the most comprehensive analysis of the host/pathogen interface of the alphaviral replication complex.

Finally, yeast two-hybrid studies have also been used to assess the P:P interactions of the alphaviral nsPs. As described in Bourai et al. (2012), several nsP proteins from CHIKV were evaluated revealing a series of interconnected interaction clusters amongst the nsP2, nsP3, and nsP4 proteins of CHIKV [69]. As to be expected, these efforts revealed novel and previously observed interactants when compared to the studies described above.

That the above efforts have led to the identification of common and unique interactants is unsurprising. It is likely that alphaviruses have evolved to utilize shared components of the host machinery to complete the molecular life cycle. Furthermore, it is equally likely that the above efforts have failed to detect all interactions equivalently, perhaps due to differences in approach and methodology, and therefore absence of a particular factor across one or more datasets is not evidence indicative of a lack of conservation. It is curious that all the above efforts have failed to specifically examine the P:P interactions of the alphaviral nsP1 protein. This is likely due to the molecular nature of the nsP1 protein, which unlike other alphaviral nsPs is palmitoylated and anchored to the host membrane. It remains probable that some host factors that interact with the nsP1 protein were detected following the purification of the SFV replication complexes [68]. Nonetheless, as no overt efforts to identify the P:P interaction network of the alphaviral nsP1 protein have been undertaken, there remains a significant gap in the knowledgebase regarding the full extent of the nsP host/pathogen interface.

Together, the above efforts to discern the P:P interactions of the alphaviral nsPs have led to several important contributions to the understanding of alphaviral molecular biology. Indeed, efforts to understand the importance of these host/pathogen interactions were a central component of many of the aforementioned studies. Prominent amongst these contributions is the realization that nsP3 engages with numerous host/pathogen P:P interactions, most notably evidenced by the identification and characterization of G3BP1 and GSBP2 [70,71,72,73,74]. Many adaptor proteins, such as the 14-3-3 family members detected during efforts involving nsP3 and nsP4, many ribosomal proteins, protein chaperones, and cytoskeletal components were also observed as being common interactants [65,68,69,70]. In addition to these other conserved host factors, a sizeable number of host RNA binding proteins were observed during these efforts, including many hnRNP proteins, several of which were further examined using RNAi-based approaches [68,69]. For many of these hnRNP proteins, RNAi silencing caused different effects on infection with respect to the individual target protein and the alphavirus being tested, thus it is possible that alphaviruses have evolved to use the same proteins to different ends during infection [68,69]. Nevertheless, whether these effects are attributable to silencing host RNA binding proteins with known roles in normal cellular function, or a specific loss of the host factor on alphaviral infection is not distinguishable through the approaches employed at the time.

### 3.2. Defining the RNA:Protein Interaction Interface of the Alphaviruses with their Host

While host/alphaviral P:P interactions have been extensively studied, there have been fewer comprehensive discovery studies that have examined the direct interactions of host proteins and the alphaviral RNAs. The renewed interest in examining alphaviral R:P interactions is due to their recognition as an important host/pathogen interface, and to improvements to the available technology to allow for RNA focused discovery approaches. As an excellent review of the many methodologies available for the study of viral R:P interactions has recently been published [75], we will not extensively delve into the individual methodologies themselves, but rather focus on summarizing studies where these approaches have been specifically used to assess alphaviral R:P interactions.

One of the earliest alphaviral R:P discovery studies utilized an unbiased cross-linking approach to isolate R:P complexes from infected cells. As reported in LaPointe et al. (2018), the alphaviral R:P interactome was probed by using a cross-link-assisted mRNA purification (CLAMP) assay approach to determine what host factors were interacting with the alphaviral RNA. This approach relied on formaldehyde cross-linking followed by vRNA specific affinity purification to identify host factors associated with the vRNAs during infection. This study led to the identification of approximately 70 host RBPs as putative interactants, and the study went on to confirm three hnRNP protein interactions, namely hnRNP K, I, and M, via UV cross-linking and immunoprecipitation [76]. To further characterize these interactions, CLIP-seq was employed to identify the R:P interaction sites, and a reverse genetics approach was used to make silent mutations in the mapped interaction sites. This approach disrupted hnRNP:vRNA binding, which reduced viral titer and intriguingly increased structural gene expression. This work was also later expanded by Gebhart et al. (2020) where the CLAMP approach was used to identify common interactants of the vRNAs of SINV, CHIKV, and VEEV [77]. Altogether, 108 common host proteins were found to associate with the vRNAs of the three different alphaviruses, of which 26 host factors were found to be described as having direct roles as RBPs. Coimmunoprecipitations of SINV, CHIKV, and VEEV vRNAs using antibodies for three different common protein interactants, hnRNP K, hnRNP A1, and ANP32A, was used to confirm these observations. Interestingly, it was found that even though these viruses share common host protein interactions, they likely do so with different affinities [77].

To date, most studies have focused on host/pathogen interactions from the viral perspective, as in what host proteins are interacting with alphaviral RNAs. However, this ignores the potential impact that alphaviral infection likely has on RNA binding proteins overall. To remedy this deficiency, the Castello lab undertook a very important study to identify system wide changes in RBPs due to SINV infection [78]. To this end, the study done by Garcia-Moreno, Noerenberg, and Ni et al. used a method called RNA-interactome capture to determine what RBPs were important during SINV infection compared to non-infected cells. This method used a mixture of isotype labeling during infection and UV crosslinking to identify proteins that interact with poly(A)-positive RNAs during infection. After cell lysis and oligo(dT) capture, the samples were quantified by proteomic analysis. The authors found that around 250 total RBPs that have ‘differential’ binding during SINV infection, inferring that the “RBPome” is significantly perturbed during alphaviral infection [78]. While the authors did find novel RBP interactions through this approach, they did not identify several previously found RBPs that have emerged during other studies. This observation is understandable, primarily for the same reasons stated above during the discussion of alphaviral P:P interactions; discovery approaches may only be interpreted as detecting and adding to the potential repertoire of interactants, as failure to detect a given factor is not evidence of absence.

While the field has begun to undertake a series of very important efforts to identify the host/pathogen RNA:Protein interface, the characterization of RNA:Protein interactions in the context of RNA-binding remains relatively new for the field. Accordingly, there has been limited descriptions and attributions of the specific functions of RBPs during alphaviral infections, and of these the known and unknowns are extensively discussed in the next section.

## 4. Ontological Analysis of Alphavirus—Host Protein Interactants

**Figure 3:**
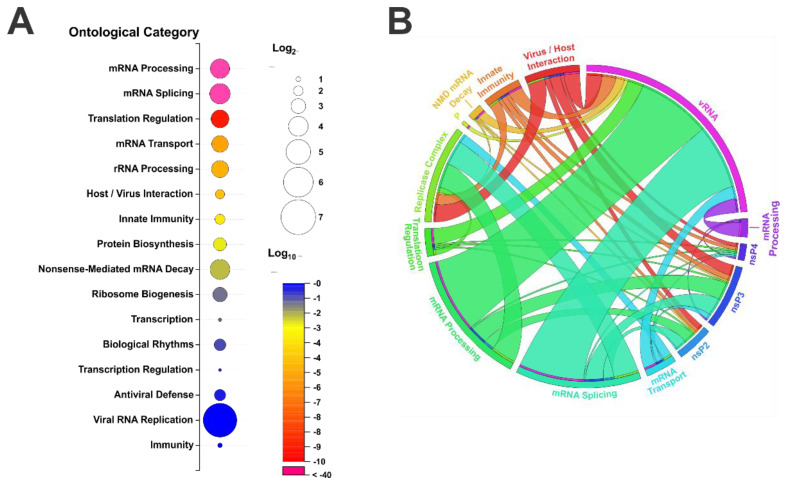
Ontological Analysis of RNA-Binding Proteins Implicated as Alphavirus Interactants. (**A**) The 125 host proteins identified as RNA-binding proteins via analysis of molecular function were assessed using DAVID to determine enriched biological processes. Shown is an enrichment plot of the top statistically enriched categories. Circle size is indicative of enrichment (relative to expected) and color indicates statistical significance (as per Bonferroni adjusted p-Values). (**B**) A chord plot of the top nine statistically enriched ontological biological processes, and the viral components with which the constituent interactants were associated. The category labeled P is abbreviated from Protein Biosynthesis.

Altogether, the above discovery efforts have led to the identification of over 600 host proteins as putative interactants with the alphaviruses, whether they interacted with the RNA, the individual components of the replicase complex, or with the replicase complex as a whole. Of these host factors, 125 of these proteins are recognized as RBPs as per DAVID ontological analysis of molecular function. As a goal of this review is to identify commonalities amongst the alphavirus associated RBPs in hope of discerning the potential role(s) they play during infection, these 125 host RBPs were further assessed using ontological analysis of biological process. As shown in Figure 3A, the top nine enriched biological processes with statistical significance after multiple test corrections include mRNA processing, mRNA splicing, translation regulation, mRNA transport, rRNA processing, host-virus interaction, innate immunity, protein biosynthesis, and nonsense-mediated decay. As many of the RBPs associated with alphaviral infection have been determined to interact with many viral components, chord mapping was performed to further determine if the interactant RBPs associated with the enriched biological process categories clustered with individual viral components (Figure 3B).

## 5. Specific Roles of RBPs during Alphavirus Infection

While there are decades of discovery efforts examining the identity and roles of host proteins involved in alphaviral infection (for a complete list see Supplemental Table S1), the importance of RBPs to alphaviral infection has only recently become the target of significant interest. Even though many host factor interactants have been identified as key contributors to alphaviral infection, relatively few of them have been examined in the specific context as RBPs to viral RNAs. As stated above, an additional primary goal of this review is to summarize the known contributions of RBPs that have been targets of study and to identify where further work is needed to fully understand their importance as RNA-binding proteins. As such, the 14 host factors that have been most thoroughly characterized are described in detail below.

### 5.1. RNA Binding Proteins

#### 5.1.1. La: A Possible Replication Regulator of 3′ Minus Strand

In the early 1990′s, efforts from the Strauss lab observed that several cellular proteins interacted with SINV RNAs and found that several host factors bound to the 3′ end of the Minus Strand RNA of SINV [62,63]. As reported in later work by Pardigon et al. (1996), one of these host proteins was determined to be the cellular La protein [79]. The La protein was hypothesized to be important for SINV RNA replication as it normally binds to the 3′ end of host transcripts and regulates transcription; however, unfortunately, further studies evaluating the role of the La protein could not be completed due to exceptionally high intracellular concentrations of the La protein (in excess of 40 nM) preventing complete silencing. Furthermore, knockout of the La protein is impossible as the protein is essential during embryogenesis [80]. Thus, despite being the first recorded instance of a host RBP affecting viral RNA function, much is still unknown regarding the importance of La to infection.

#### 5.1.2. HuR/ELAV1: Stabilizing the Alphaviral RNAs from 3′ RNA Decay

The HuR/ELAV1 proteins, and its closely related homologs with tissue specific expression patterns, are RNA recognition motif (RRM) containing proteins that have been identified as potent regulators of RNA stability and gene expression [81,82]. RNA stability conferred by HuR binding is imparted by the direct interactions of the HuR protein with poly(U)-rich (URE) and AU-rich elements (AREs) found in the 3′UTRs of cellular transcripts [82,83,84,85].

Near the turn of the century, the Wilusz lab, using a series of tissue culture model systems and biochemical approaches, found that the host protein HuR binds to the 3′ UTR of alphaviruses to stabilize them for successful infection [86,87]. During infection, HuR will bind to either the RSE, the URE, and/or the CSE depending on the alphavirus, and prevent deadenylation to keep alphaviral mRNAs from being degraded [88]. While HuR binds to the URE region of SINV, the CSE region is needed for binding of RRV and CHIKV as well as the third RSE for CHIKV [89]. Normally, HuR is in the cytoplasm in mosquito cells; however, it is located in the nucleus in mammalian cells and relocates to the cytoplasm after dephosphorylation during SINV infection. Relocalization of the HuR protein is not due to cellular stress, but is a response specifically found in alphaviral infections as infection with Measles or Dengue viruses did not cause relocalization. The HuR protein is conserved in both mosquitoes and humans, and both homologs have been found to associate with similar elements in the alphaviral 3′UTRs [87]. This conservation indicates that HuR binding could be important for infections across multiple host systems, and knockdown of HuR or the deletion of the binding site in the SINV 3′UTR caused a reduced viral titer in both mammalian and mosquito tissue culture models.

#### 5.1.3. G3BP1/2: Regulation of Alphaviral Replicase and Minus Strand Synthesis

Ras GTPase-activating protein-binding proteins 1 and 2 (G3BP1/2) are critical for the formation of stress granules due to multiple different environmental stressors, including viral infection [90,91,92]. These proteins form homo- and hetero-multimers with each other to induce stress granule formation [93]. G3BP1 and G3BP2 are homologous and have some redundancy in alphaviral infection; therefore, in most studies, they are collectively referred to as G3BP. Studies examining the roles of the G3BP proteins during alphaviral infection have been a prolific area of study, and as such, the G3BPs have been found to interact with the nsP3 proteins of around 17 different alphaviruses and also replicase proteins nsP2 and nsP4 [65,66,70]. Studies performed by Cristea et al. in the MacDonald lab found that the knockdown of G3BP caused viral nonstructural protein expression and titer to be significantly increased starting early in infection and kept the trend through late infection [70]. However, knockdown only slightly effected RNA levels, but not enough to attribute the change in titer. The mosquito homologue of G3BP is Rasputin (Rin), and knockdown of Rin also did not significantly affect CHIKV RNA levels during infection [94]. To determine at what point G3BP is affecting the viral lifecycle, Scholte et al. (2015) determined that G3BP was not linked to CHIKV entry or nonstructural protein translation but was involved the switch to negative strand synthesis [72]. The McInerney lab comprehensively studied the effect of G3BP knockdown on several alphaviral nsP3s interactions and found that for most OW alphaviruses replication and transcription were reduced in the absence of G3BP [74]. Further characterizing this interaction, the authors found a link between nonstructural polyprotein processing and G3BP dependence. Overall, they concluded that G3BP is proviral at several points through the alphaviral lifecycle, as it most likely is involved in activation of the replicase and negative strand synthesis, but not the switch from RNA translation to replication [71,74]. Despite G3BP’s role in binding to the alphaviral replicase complex being a subject of extensive investigation, the role of G3BP specifically as an RBP is largely overlooked. While G3BP may be involved in RNA replication and minus strand synthesis, it is intimately linked to the replicase as a complex. At this point, with the limitations of the current technology, it would be nearly impossible to distinguish the impact of G3BP on RNA binding without disrupting the viral replicase.

#### 5.1.4. TIA1/R: Promotes Infection by Forming Stress Granule Decoy

T-cell-restricted intracellular antigen 1 (TIA1) and TIA1-Related (TIAR) proteins regulate RNA splicing, protein translation, and stress granule formation [95,96,97]. These proteins are included into initiation complexes, which then collect and sequester these initiation components away in SGs in response to environmental or intracellular stress stimuli. During alphaviral infection, SGs are disassembled in the vicinity of viral replication complexes despite viral infection inducing a clear state of cellular stress [98]. McInerney et al. (2005) explored the importance of these proteins during SFV infection and found that TIA1/R diffused away from SGs throughout the cell when viral replication was active. In TIA1 knockdown MEF cell lines, host cell translational shutoff was delayed, and early SG formation was decreased. This led the authors to conclude that TIA1/R forming SGs at the beginning of viral infection was fulfilling a pro-viral role as it removes cellular mRNAs so the subgenomic vRNAs do not have to compete for translation with the active polysomes [98]. Although TIA1/R have RNA binding abilities and are involved in RNA splicing, RNA binding roles on alphaviral RNAs have not been evaluated.

#### 5.1.5. GEMIN5: Regulation of Protein Expression through 5′ UTR Interaction

Gem-associated protein 5 (GEMIN5) is an RBP that catalyzes the formation of the spliceosome, can bind to the 7-methylguanosine cap of RNA strands, and can control protein synthesis by interacting with the ribosome [99,100,101]. Garcia-Moreno et al. (2019) found that GEMIN5 was significantly stimulated by SINV infection and colocalized with viral factories [78]. Over-expressing a GEMIN5-EGFP fused protein caused a delay in viral subgenomic gene expression and inhibited capsid expression. While GEMIN5 binds to the 3′ UTR of some host mRNAs, this RBP was found to interact with the 5′ ends of both SINV RNA species. GEMIN5 effects viral protein expression through this interaction at the 5′ end; however, GEMIN5′s role as an RBP has not been characterized in depth.

#### 5.1.6. FXR1: Interaction with the nsP3 Hypervariable Domain Promotes Infection

The Fragile X-Related Protein 1 (FXR1) is involved in post-transcriptional mRNA regulation [102]. After discovering FXR1 that interacts with SINV nsP3, the Frolova/Frolov group continued to functionally characterize this interaction [64]. The Protein:Protein interaction of FXR1 mapped specifically to the VEEV nsP3 Hypervariable Domain (HVD) within nsP3, and FXR1 moves from the nucleus to the viral replicase complexes during infection [73]. Knockout of the FXR1 family only reduced VEEV viral growth titers while unaffecting the OW alphaviruses SINV and CHIKV. Addition of these proteins reinstated viral titer almost back to WT like levels, which indicates that the FXR1 family is important for VEEV infection. Removal of the interaction site of FXR1 in the HVD region of nsP3 causes diminished pathogenesis of VEEV and EEEV [103,104]. Further elucidating this interaction, the authors found that FXR1 is important RNA synthesis and replicase complex formation. This interaction also holds true for EEEV, albeit the interaction is a separate distinct site. Direct impact of FXR1 as an RBP has not been well characterized in alphaviral infections.

#### 5.1.7. DEAD-Box Helicases: Promotion of Spreading Infection to Neighboring Cells

The family of DExD/H-box RNA helicases are involved in many RNA processing roles like splicing, transcription, RNA export, translation, stress granule formation, and innate immune sensing [105,106,107]. As such, many of these helicases have been isolated during alphavirus interaction studies. In studies performed by Amaya et al. (2016), they found that DDX1 and DDX3 interacted with VEEV nsP3 [108]. The removal of DDX3 and DDX1 by siRNA knockdown reduced the viral titer and intracellular and extracellular viral RNA. It is interesting that the decrease of intracellular viral RNA seemed to be more significant at earlier time points in the infection whereas extracellular viral RNA had a more considerable decrease at 24hpi. This observation was not as evident in the double DDX1/DDX3 knockdown; however, the same trends of decreased viral titer and RNA levels late during infection was evident. From these data, the authors conclude that the role of these helicases are important during multiple rounds of infection, due to the low amount of virus inoculated and the decreasing trends becoming more significant at later times during infection [108].

RNA helicase DHX9 is known to unwind DNA and RNA structures and has been implicated during many other viral infections including picornaviruses, orthomyxoviruses, pestiviruses, flaviviruses, and retroviruses [109,110,111,112]. Studies completed by Varjak et al. (2013) and Matkovic et al. (2019) both show that DHX9 interacts with the SFV and CHIKV replicase complex, respectively [68,113]. Matkovic et al. (2019) further show that DHX9 relocates to viral replicase complexes during infection, and knockdown of this helicase promotes infection while overexpression of DHX9 inhibits infection [113]. DHX9 also negatively regulates both positive and negative RNA strand synthesis. Since DHX9 can sense dsRNA, it was hypothesized that DHX9 is involved in innate immune sensing and establishment of an anti-viral state. However, IFNβ levels were not affected during knockdown of DHX9 by CRISPR/Cas9 editing. Interestingly, knockdown reduced genome translation while overexpression enhanced translation of the viral nsPs. Enhancement of translation but overall reduction in infection with the lack of DHX9 points to this protein having a role during alphaviral infection; however, a specific role as an RBP has not been characterized [113].

### 5.2. Heterogenous Nuclear RibonucleoProteins (hnRNPs)

Many individual proteins make up this diverse family of RBPs, and they are all heavily involved in RNA regulation, often over multiple biological processes [114]. Individually and altogether, they have roles that include splicing, mRNA stability, translational and transcriptional regulation, and mRNA decay. Due to the presence of nuclear localization signals, the majority of the hnRNPs are located in the nucleus; however, they can shuttle to the cytoplasm for various reasons during their normal life as cellular RBPs. Some hnRNPs can be post-translationally modified which in turn changes their binding preferences, molecular functions, or subcellular locations. Over the years, many hnRNPs have been implicated not only in alphaviral infection but also many other viral infections as well.

#### 5.2.1. hnRNP A1: Promotion of RNA Replication and Synthesis

This protein is part of the hnRNP A/B subfamily. In addition to hnRNP A1 having been detected in the majority of the alphaviral interaction discovery efforts, it has been shown to promote infection of RNA and DNA viruses such as mouse hepatitis virus, hepatitis C virus (HCV), dengue virus, human papilloma virus 16 (HPV), and vesicular stomatitis virus [115,116,117,118,119]. Regarding the alphaviruses, studies performed by Lin et al. demonstrated that hnRNP A1 relocates from the nucleus to the cytoplasm during infection where it interacts specifically with the 5′ UTR of the SINV gRNA [120]. The knockdown of hnRNP A1 by siRNA reduced viral gene expression, vRNA synthesis, and viral titer to a significant extent. Continued work out of Mei-Ling Li’s lab determined that hnRNP A1 interacts with both the genomic and subgenomic promoters of SINV positive strand [121]. Gui et al. went on to suggest that the removal of this protein from host cells reduces genomic and sgRNA synthesis, and the addition of hnRNP A1 in knockout cells can rescue the vRNA synthesis.

#### 5.2.2. hnRNP C: Negative Regulator of Alphaviral Infection

Regulatory molecular switches like m6A help hnRNP C bind to the mRNA and regulate splicing events and mRNA stability during normal host biology [122]. Efforts undertaken by Varjak et al. (2013) characterized the impact of hnRNP C on alphaviral infection. During hnRNP C knockdown, viral genomic and subgenomic protein expression was increased as compared to control cells infected with SFV, CHIKV, and SINV [68]. In addition to hnRNP C’s effects on translation, viral titer and vRNA synthesis were increased as well. Altogether, it is interesting that removal of hnRNP C allowed for better alphaviral infection, when during normal biology it is involved in cellular mRNA stability. Thus, for alphaviruses at least, hnRNP C expression negatively influences viral infection. Along a similar vein, hnRNP C is also implicated in other viral infections, where it seems to have mixed uses, as during alphaviral infection. The knockdown of hnRNP C increased adenoviral protein expression and viral titer; however, during Dengue virus infection, removal of hnRNP C was detrimental to viral replication and protein expression [123,124]. Although hnRNP C bound to alphaviral RNA has not been studied, hnRNP C interacts with the negative strand of poliovirus to promote positive strand synthesis [125].

#### 5.2.3. hnRNP K: Phosphorylated Form Regulates RNA Synthesis and Gene Translation

The hnRNP K protein has three KH domains, which allows this protein to be involved with RNA regulation and processing and bind to DNA to regulate transcription [126]. The hnRNP K protein interacts with many different host proteins and has a wide array of functions in the nucleus and cytoplasm. Since the finding that hnRNP K interacts with the replicase complex in SINV infection by Burnham et al. (2007), hnRNP K has been found to interact with alphaviruses in every discovery effort since [127]. Differentially phosphorylated states of hnRNP K were both found to have been increased in the cytosolic fraction after SINV infection, though mostly the phosphorylated form interacts with the viral replicase machinery containing the sgRNA strand. Silencing of hnRNP K also decreases the number of SINV infected cells at 6hpi, indicating an important role for hnRNP K in the early stages of viral infection. In studies performed by Bourai et al. (2012), the authors knocked down CHIKV nsP2 interactants, which included hnRNP K [69]. In this study, knockdown caused decreased viral gene expression over time, and a lower viral titer of CHIKV. Varjak et al. (2013) found that hnRNP K knockdown inhibited CHIKV and SINV infection by decreasing protein expression [68]. Interestingly, Varjak et al. (2013) reported that the knockdown of hnRNP K did not affect SFV viral titer and RNA synthesis over time, and did not significantly affect SFV viral protein expression until 8hpi, which correlated with the localization of hnRNP K to areas of replication. Localization has not been characterized before 6hpi, so the precise timing as to when hnRNP K, as well as other hnRNPs, moves early in infection is unknown.

Unlike many of the other proteins reviewed here, the hnRNP K protein has been evaluated regarding its capacity to bind to and influence viral RNAs during infection. Specifically, as reported in LaPointe et al. (2018), the hnRNP K protein interacts with a distinct interaction site on the subgenomic vRNA [76]. Disrupting this site with the incorporation of silent mutations through the primary binding site caused lower viral titer in mammalian cells and intriguingly increased structural protein expression. Both studies validate the previous findings that hnRNP K is beneficial for Old-World alphaviral infection. Overall, hnRNP K regulation most likely depends on the phosphorylation status of the protein, and which alphavirus it interacts with.

#### 5.2.4. hnRNP M: Regulation of RNA Synthesis and Translation

The hnRNP M protein has been indicated to be involved in the splicing of immune gene transcripts, and it has been shown to dampen the innate immune response during some RNA viral infections by blocking host vRNA sensing receptors [128,129]. As with the hnRNP K protein, hnRNP M was identified as a component of the SFV replicase complexes and was shown to colocalize with the viral replicase machinery [68]. Silencing hnRNP M increased viral gene expression during SFV, CHIKV, and SINV infection, although the effect was minimal with the Old-World alphaviruses. Viral titer was also increased during hnRNP M knockdown as compared to WT SFV infection. Nonetheless, hnRNP M knockdown only mildly effected RNA synthesis by increasing RNA levels at 8hpi.

As with hnRNP K, the specific binding affinity of the hnRNP M protein have been directly evaluated. Indeed, reports from LaPointe et al. (2018) have found that hnRNP M directly interacts with the vRNA, primarily with the subgenomic strand [76]. As with hnRNP K, disrupting the interaction site by mutating the vRNA caused a decrease in hnRNP M binding, inhibition of viral growth kinetics, and an increase in structural protein expression.

LaPointe et al. (2018) reported some opposing results from the previously discussed results from Varjak et al. (2013). After disruption of the interaction site, the loss of binding caused a reduction in viral titer in both mammalian and mosquito tissue culture models, although no effect on vRNA synthesis was observed [76]. Viral protein expression, specifically that of the structural proteins, was significantly increased. These opposing results could be because the LaPointe studies used an interaction disruption approach, whereas the Varjak study used an RNAi approach to knockdown hnRNP M expression, whcih could have impacted cell biology beyond viral infection, resulting in off-target or indirect consequences on viral infection [68]. While the precise reason underlying this discrepancy is unknown, we argue that disrupting the interaction while leaving host biology unperturbed allows for a more direct conclusions to be made regarding the importance of RBPs to viral infection.

#### 5.2.5. PCBP1/hnRNP E1: Unknown Promotion of Alphaviral Infection

Along with hnRNP K, hnRNP E1 and E2 are the only hnRNPs that have an RNA binding KH domain [130]. The hnRNP E1 has been shown to be involved in translational control of viruses like poliovirus, HPV and HCV [131,132,133]. In 2013, Varjak et al. found that PCBP1 does not colocalize with SFV nsP3 proteins; however, it is still important for infection [68]. During PCBP1 knockdown, viral gene expression was negatively impacted. Viral growth kinetics were also decreased compared to WT SFV infection. Silencing of hnRNP E1 caused a slight reduction in RNA synthesis early at 4hpi; however, this effect seemed to disappear later in infection. Infections with CHIKV and SINV also continued the trend of lower luciferase expression when hnRNP E1 levels were reduced. These data led the authors to conclude that hnRNP E1 supports alphaviral infection. However, there are no known experiments studying the effect of overexpression of this protein, and there is no data on the role of hnRNP E1 as an RBP.

#### 5.2.6. PTBP1/hnRNP I: Interaction with vRNA Regulations Translation

PTBP1, also known as hnRNP I, plays a role in alternative splicing and can regulate RNA stability, replication, and translation [134]. hnRNP I preferentially binds to polypyrimidine tracts by the four RNA binding domains and each domain has a high affinity for sequences containing 15 to 25 pyrimidines [135]. The third and fourth binding domain of hnRNP I can even bind the same RNA and remodel the structure to form a loop [136]. As well as alternatively splicing host mRNA, most of the functions of hnRNP I are enhancing RNA stability and translation by binding and preventing RNA degradation [134]. Not only does hnRNP I promote host mRNA translation, it has also been shown to bind to the picornavirus internal ribosome entry site (IRES) and stimulate translation [137,138]. As well as for picornaviruses, hnRNP I has been implicated in several viral infections including being upregulated during ZIKV infection, interacting with several coronaviruses, regulating hepatitis B virus, and interacts with the influenza viral protein NS1 [139,140,141,142,143].

Studies by the Sokoloski lab have characterized hnRNP I interactions with alphaviruses as their roles as an RBP. Like the other characterized hnRNP interactions with SINV, the hnRNP I protein interacts with SINV subgenomic vRNA; however, its primary binding site is in the 3′ UTR region [76]. Curiously, the SINV hnRNP I interaction site differs from the canonical binding motifs observed in cellular transcripts, suggesting that hnRNP I (and likely other RBPs) may have altered binding affinities during infection [136]. The deletion of this interaction site from the vRNA causes diminished protein binding and decreased viral growth in mammalian and mosquito cells lines [76]. Despite having a lower viral titer and somewhat of a downward trend of vRNA synthesis late during infection, viral structural gene expression significantly increased. Recently, work published in Westcott et al. elucidated the role of hnRNP I during alphaviral infection by employing a protein tethering strategy [144]. It was found that restoring the hnRNP I interaction with the SINV RNA restores viral titer, structural protein expression, and viral particle function. Interestingly, disrupting the hnRNP I interaction site caused an apparent molecular bottleneck, which resulted in the disruption of glycosylation of the viral glycoproteins. This in turn caused poor particle function, which was restored by reestablishing the protein interaction.

## 6. Perspectives and Limits of Understanding

### 6.1. Picking a Dance Partner: Who or What Decides Viral RNA Binding Specificity?

While host protein interaction studies have started to focus on specific RBPs and their interactions directly with the bound viral RNAs, an interesting question is raised when multiple vRNAs are expressed from a single template: How might colinear RNAs have different binding repertoires despite having identical sequences? For example, studies from several different labs have found that different RBPs like hnRNP K, hnRNP I, and hnRNP M all preferentially bind to the subgenomic vRNA in distinct interactions to promote alphaviral infection, despite the gRNA and sgRNA having them being the same sequence [76,127]. The La protein, one of the first host proteins to be found to interact with vRNA, preferentially binds the 3′ end of the negative strand to likely promote viral replication [63]. Additionally, at the 3′ region, HuR binds to the URE and promotes RNA stability [86,87]. At the opposite end, hnRNP A1 is involved in translation by binding to the 5′ UTR of genomic vRNA [120,121]. While it is unknown exactly why these interactions are specific to a particular RNA strand, we can hypothesize that RBPs preferentially bind to the vRNA depending on the RBP function or even the RNA function as well. In contrast with the sgRNA that does translate, a large part of the genomic vRNA strand and the minus strand do not translate; therefore, it stands to reason that they may have very different repertoires of RBPs. Additionally, the specificity conundrum may also be solved through the existence of dual occupancy interaction sites, such as that posed by the SINV capsid protein and the host hnRNP M protein [76,145].

### 6.2. Dancing Backwards and in High Heels: Differential Host Functions and Requirements

As demonstrated by a large body of data, RBP interactions are important during mammalian infection, but there are also many RBP homologues in other hosts of alphaviral infection, like mosquitoes, that are equally or differentially important. Proteins like HuR, the G3BP homologue Rasputin, hnRNP A1, and PTBP1 all have been characterized during infection of mosquito cell culture models [86,87,94,120,121,144]. These studies have varied in whether they utilized knockdown or disrupted protein binding by mutating the interaction site sequence, but perturbing the RBP or the RBP interaction almost always negatively affected alphaviral infection. The effects seen during mosquito cell line infection reflect the same effect in mammalian tissue culture, and the conclusions from the vertebrate system are assumed to be true for the invertebrate. However, it is not known if all RBP interactions have the same role in multiple hosts or if there are host dependent RBP interactions; therefore, it is important to not apply the same conclusion to each system.

On a similar note, within a single host system there can be considerable variation regarding RBP expression levels and patterns. This reality creates a patchwork of host systems at the cellular level that may, or may not, support viral replication should a critically necessary RBP be present or absent. A consequence of this may be tissue or cellular restriction, in addition to the possibility that the viral lifecycle is differentially regulated in different host cell systems.

In addition to the diversity of RBPs from a host and cellular perspective, there is notable evidence that individual RBPs may have different impacts on alphaviral infection in a viral species dependent manner. For example, the hnRNP K protein has been described to have differential impacts on alphaviral replication across SINV, CHIKV, and SFV infections. As such, it is difficult to uniformly extend observations regarding the impact of specific RBPs across multiple alphavirus species. Thus, while it is safe to say that an individual RBP is a conserved interactant of many alphavirus species, broad statements regarding specific contributions to viral infection may be prone to inaccuracy. Thus, there is an ongoing need for research that defines the specific importance of RBPs to viral infection across all potential host systems and across the different alphaviral species.

### 6.3. Moving to the Beat: Subcellular (Re) Locations of RBPs during Infection

In most cases, the roles of the RBP during infection and the interaction themselves are time dependent. During normal cellular function, the majority of the RBPs, especially the hnRNPs, spend the better part of their time in the nucleus, and are shuttled back and forth to the cytoplasm during their normal functions in host RNA biology [114]. During infections with alphaviruses, many of these RBPs shuttle out of the nucleus and stay in the cytoplasm around replication complexes for the remainder of infection [68,119,120,121,127]. Many also have certain roles either early or late during infection, as experimental time courses have discerned the difference. The relocalization of RBPs during infection could be due to several factors including post-translational modifications like phosphorylation.

Proteins like HuR and hnRNP K have been shown to have different roles and localizations depending on the differential phosphorylation states of the proteins. During normal biology, HuR can be phosphorylated, which is associated with the localization of the protein [82]. A study by Dickson et al. (2012) shows that during SINV infection, HuR is dephosphorylated, and it is hypothesized that this causes the protein to relocate from the nucleus to the cytoplasm during infection, in which the vRNA sequesters HuR away like a sponge [89]. Both forms of unphosphorylated and phosphorylated hnRNP K were found in the cytosol during SINV infection, although mostly the phosphorylated form interacted with the replicase machinery and subgenomic vRNA [127]. It has been shown that hnRNP K has multiple phosphorylation sites, and these are all important for the different functions and locations of hnRNP K [146]. While we do know that hnRNP K is phosphorylated during infection, we do not know the specific role the phosphorylated protein plays [127]. In normal host biology, phosphorylation states can determine the role of RBPs, almost like a switch, and effects binding to RNA or shuttling out of the nucleus [146]. These differential states most likely also affect alphaviral infection, although they have not been described or studied in detail.

### 6.4. Taking Center Stage: Perspective Roles of RBPs during Infection

One of the biggest questions that arises from studies of host proteins during alphaviral infection is whether these interactions are pro- or anti-viral. Unfortunately, despite these interactions being researched for decades, the answers to this question are extremely nuanced. Depending on the study, the effect of the RBP on the alphavirus, and the way of inhibiting the RBP during viral infection, the impact of the RBP could be interpreted as being either pro- or anti-viral. For instance, hnRNP U has been shown to be antiviral and activate type-I IFNs during infection with DNA and RNA viruses, although this specific interaction has not been shown for alphaviruses [147]. In Varjak et al. (2013), the knockdown of hnRNP M proved to be beneficial by increasing viral genomic and subgenomic expression and viral titer; however, studies from our lab have shown that disrupting the interaction with vRNA causes decreased viral titer, albeit while increasing structural protein expression [68,76]. Thus, while both studies concur that hnRNP M is important to infection, the precise nature of the interaction to alphaviral infection is nuanced. There may also be roles for the hnRNPs to repress innate immune sensing as it binds to vRNA sensing proteins and it represses splicing and processing of immune transcripts [128,129]. In most studies, RBPs act in a pro-viral way since interrupting the interaction causes decreased viral growth along with other phenotypes as described above. The dual evolution of host protein and alphaviral interactions have caused an already complex system to become more multifaceted. Alphaviruses have most likely hijacked the host system to benefit infection, although some RBPs still may have innate or intrinsic antiviral properties.

## Data Availability

All data pertinent to this manuscript may be found in the accompanying supplemental materials.

## Supplementary Materials

The following supporting information can be downloaded at: https://www.mdpi.com/article/10.3390/v15010164/s1. Table S1: Interactant List Tables and Ontological Assessments.
